# Supplementary material for: Annual Crops Contribute More Predators than Perennial Habitats during an Aphid Outbreak
Source: Insects. 2023 Jul 11;14(7):624. doi: 10.3390/insects14070624 (PMC10380491; doi:10.3390/insects14070624)
Supplement: Supplementary file 1 [file insects-14-00624-s001.zip › insects-2481314-supplementary.pdf]

Supplementary materials

Table S1. Field locale, the latitude and longitude of each soybean field site, field size in hectares (ha), the adjacent habitat where bi-directional Malaise traps were placed, and the vegetative (V) and reproductive (R) stage of soybean from field plant counts (n=20) in 2017. For growth stages with NA's, plant counts were not conducted in these fields. Woody vegetation included saskatoon (*Amelanchier alnifolia* (Nutt.) Nutt.) and chokecherry (*Prunus virginiana* L.) bushes, a grassy understorey, and an overstorey dominated by deciduous trees, mainly white poplar (*Populus* spp.), oak (*Quercus* spp.), Manitoba maple (*Acer negundo* L.), and American elm (*Ulmus americana* L.).

**Field name**	**Latitude**	**Longitude**	**Adjacent habitat**	**Median soybean growth stage**		
				**Week 0**	**Week 1**	**Week 2**
bar7	50°34'01.9"N	97°00'32.7"W	Woody vegetation	V6, R2	V8, R2	V7, R2.5
col7	50°22'13.4"N	96°27'04.4"W	Alfalfa	V7, R2	V8, R2	V9, R3
cra7	49°20'57.1"N	97°25'56.4"W	Canola	NA	V10, R3	V10, R4
dea7	49°54'15.3"N	98°00'01.9"W	Spring wheat	NA	V7, R3	V9, R2
den7	49°01'45.3"N	97°05'01.4"W	Woody vegetation	V6, R2	V9, R3	V10, R3
gle7	50°16'15.8"N	96°28'14.9"W	Alfalfa	V6, R3	V7.5, R2	V9, R3
mar7	50°00'13.5"N	96°25'08.8"W	Woody vegetation	V7, R3	V10, R2	V10, R3
mik7	49°15'23.5"N	97°07'37.4"W	Spring wheat	V5, R1	V9, R3	V9, R3
par7	49°34'13.3"N	98°01'05.4"W	Spring wheat	NA	V8, R2	V9, R3
pen7	49°38'46.8"N	97°54'30.4"W	Canola	NA	V9, R3	V9, R2
ran7	49°10'02.4"N	97°54'39.3"W	Canola	V7, R3	V9, R5	V8.5, R5
ste7	49°52'31.0"N	98°02'45.3"W	Alfalfa	NA	V8, R3	V9, R2

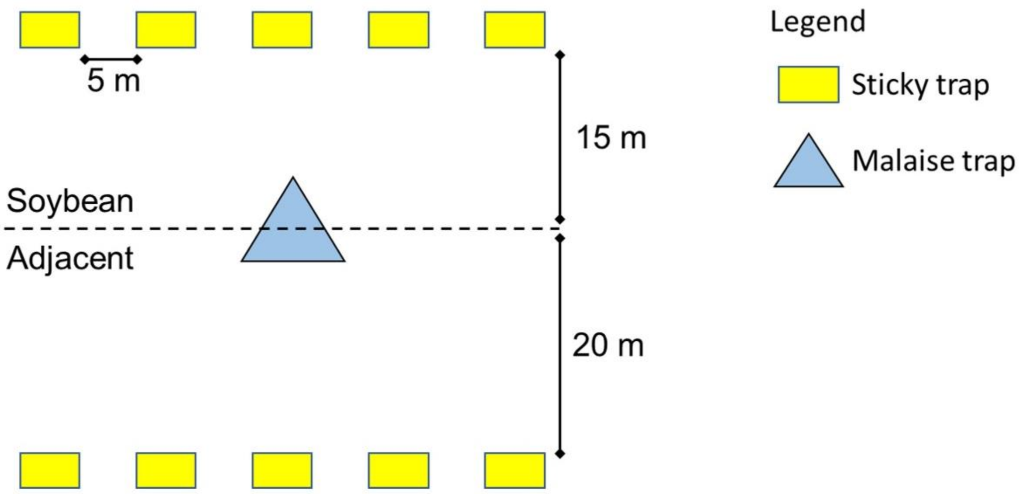

Figure S1. Sticky trap and bi-directional Malaise trap placement per field site.

Table S2. Comparing predator abundance in soybean and across adjacent habitats. The average number of individuals captured per day in sticky traps (± the standard error, S.E.) in soybean (n=12), alfalfa (n=3), canola (n=3), spring wheat (n=3), and woody vegetation (n=3). In each field, counts of the five sticky traps were summed prior to averaging across weeks and fields (n=48).

	Soybean	Alfalfa	Canola	Wheat	Woody
Coccinellids	2.57 ± 0.61	0.43 ± 0.19	0.16 ± 0.064	1.50 ± 0.31	0.48 ± 0.41
Coccinella septempunctata	0.64 ± 0.23	0.29 ± 0.14	0.071 ± 0.071	0.89 ± 0.22	0
Harmonia axyridis	1.00 ± 0.31	0	0	0	0.071 ± 0.041
Hippodamia variegata	0.22 ± 0.18	0	0	0.11 ± 0.11	0
Hippodamia parenthesis	0.094 ± 0.069	0.024 ± 0.024	0	0.024 ± 0.024	0
Hippodamia convergens	0.012 ± 0.0080	0	0	0	0
Hippodamia tredecimpunctata	0.60 ± 0.16	0.048 ± 0.024	0.068 ± 0.0030	0.47 ± 0.087	0
Coccinella trifasciata	0.012 ± 0.0080	0.048 ± 0.048	0	0	0
Psyllobora vigintimaculata	0	0.024 ± 0.024	0	0	0.40 ± 0.40
Coleomegilla maculata	0	0	0.024 ± 0.024	0	0
Syrphids [ab]	80.4 ± 17.0	8.45 ± 0.78	87.4 ± 13.1	48.2 ± 20.5	23.0 ± 20.3
Toxomerus marginatus	11.5 ± 3.61	1.40 ± 0.72	10.7 ± 1.24	3.22 ± 1.92	0.30 ± 0.044

	Soybean	Alfalfa	Canola	Wheat	Woody
All other predators combined [c]	2.45 ± 0.54	0.93 ± 0.41	1.34 ± 0.39	1.45 ± 0.78	0.64 ± 0.30
Chrysopids	0.23 ± 0.070	0.071 ± 0.00	0.024 ± 0.024	0.095 ± 0.063	0.12 ± 0.086
Chrysoperla carnea	0.19 ± 0.064	0.048 ± 0.024	0.024 ± 0.024	0.095 ± 0.063	0
Chrysopa oculata	0.030 ± 0.014	0.024 ± 0.024	0	0	0
Chrysopa chi	0.0060 ± 0.0060	0	0	0	0.095 ± 0.095
Hemerobiids	0.0060 ± 0.0060	0	0	0.024 ± 0.024	0
Anthocorids	1.69 ± 0.55	0.36 ± 0.16	0.41 ± 0.28	0.91 ± 0.74	0.024 ± 0.024
Nabids	0	0.024 ± 0.024	0	0	0
Staphylinids	0.52 ± 0.061	0.48 ± 0.23	0.91 ± 0.089	0.42 ± 0.043	0.50 ± 0.36

[a] *E. americanus* was not identified as identification by genitalia was not possible.

[b] *T. marginatus* was not identified due to low captures observed in Malaise traps.

[c] All other predators combined includes chrysopids, hemerobiids, anthocorids, nabids, and staphylinids.

Table S3. Average predator movement into soybean, movement out of soybean, and bi-directional movement (average into + out of soybean) per day, and the total number of individuals captured moving in soybean (n=12 fields). Averaged values were averaged across weeks prior to averaging across field sites.

Family<br>Species	Into soybean	Out of soybean	Bi-directional	Total captured
Coccinellidae	1.49	0.74	2.23	359
Coccinella septempunctata Linnaeus, 1758	0.96	0.52	1.48	243
Harmonia axyridis (Pallas, 1773)	0.12	0.084	0.20	35
Hippodamia variegata (Goeze, 1777)	0.0052	0	0.0052	1
Hippodamia parenthesis (Say, 1824)	0.032	0.018	0.05	8
Hippodamia convergens Guerin, 1842	0.14	0.034	0.17	28
Hippodamia tredecimpunctata (Linnaeus, 1758)	0.2	0.071	0.27	45
Coccinella trifasciata perplexa Linnaeus, 1758	0.018	0.012	0.03	5
Brachiancantha ursina (Fabricius, 1787)	0.007	0	0.007	1
Adula bipunctata (Linnaeus, 1758)	0	0.006	0.006	1
Staphylinidae	0.98	1.68	2.66	458
Syrphidae	108.92	75.43	184.35	31,174
Toxomerus marginatus (Say, 1823)	29.38	30.23	59.61	10,231
Toxomerus geminatus (Say, 1823)	1.03	0.58	1.61	270
Eupeodes americanus (Wiedemann, 1830)	71.28	40.56	111.84	18,584
Chrysopidae	0.4	0.42	0.82	134
Chrysoperla carnea (Stephens, 1836)	0.24	0.28	0.52	87
Chrysopa oculate Say, 1839	0.13	0.12	0.25	42
Chrysopa chi Fitch, 1855	0.006	0	0.006	1
Chrysopa quadripunctata Burmeister, 1839	0	0.006	0.006	1
Meleoma emuncta (Fitch, 1855)	0.006	0.0012	0.007	3
Hemerobiidae	0.24	0.14	0.38	68
Hemerobius stigma Stephens, 1836	0.15	0.077	0.23	39
Hemerobius simulans Walker, 1953	0	0.0052	0.0052	1
Micromus angulatus (Stephens, 1836)	0.03	0.024	0.054	9
Micromus subanticus (Walker, 1853)	0.055	0.031	0.086	14
Sympherobius amiculus (Fitch, 1855)	0.0052	0.0052	0.010	2
Anthocoridae	0.025	0.1	0.13	21
Nabidae	0.12	0.036	0.16	26

Table S4. Effects of movement direction (into or out of soybean), adjacent habitat type (alfalfa, canola, spring wheat, woody vegetation), and week (1 or 2) on predator bi-directional movement in soybean from a linear mixed effects model (n=48). Number of parameters (k), corrected Akaike's information criterion (AIC_c), Akaike's weights (w_i), log-likelihood (Log-lik), ratio of log-likelihood tests (L ratio), and the significance of the models compared (p-value) are presented. Models in bold were selected as the best model by AIC and log-likelihood tests.

Statistical model	k	AIC_c	w_i	Log-lik	Models compared	L ratio	*p*
Syrphids							
1) direction + week	5	62.84	0.06	-25.71			
**2) direction + adjacent + week**	**8**	**57.36**	**0.91**	**-18.83**	**1 vs 2**	**13.75**	**0.0033**
3) direction x adjacent + direction + adjacent + week	11	64.15	0.03	-17.41	2 vs 3	2.84	0.42
Toxomerus marginatus							
**1) direction + week**	**5**	**79.92**	**0.76**	**-34.24**			
2) direction + adjacent + week	8	82.29	0.23	-31.3	1 vs 2	5.89	0.12
3) direction x adjacent + direction + adjacent + week	11	89.39	0.01	-30.03	2 vs 3	2.54	0.47
Eupeodes americanus							
1) direction + week	5	76.15	0.06	-32.36			
**2) direction + adjacent + week**	**8**	**70.75**	**0.9**	**-25.53**	**1 vs 2**	**13.67**	**0.0034**
3) direction x adjacent + direction + adjacent + week	11	77.16	0.04	-23.91	2 vs 3	3.23	0.36
Coccinellids							
1) direction + week	5	40.27	0.21	-14.42			
**2) direction + adjacent + week**	**8**	**37.7**	**0.76**	**-9.01**	**1 vs 2**	**10.82**	**0.0127**
3) direction x adjacent + direction + adjacent + week	11	44.36	0.03	-7.51	2 vs 3	2.99	0.39
All other predators combined							
**1) direction + week**	**5**	**41.12**	**0.82**	**-14.84**			
2) direction + adjacent + week	8	44.65	0.14	-12.48	1 vs 2	4.73	0.19
3) direction x adjacent + direction + adjacent + week	11	47.02	0.04	-8.84	2 vs 3	7.27	0.064

Table S5. Average predator movement into and out of soybean fields adjacent to alfalfa, canola, spring wheat, and woody vegetation of the main predator groups across weeks (n=2) and study sites (n=12) from bi-directional Malaise traps. Means ± standard errors are presented.

	Alfalfa		Canola		Wheat		Woody	
	Into soybean	Out of soybean	Into soybean	Out of soybean	Into soybean	Out of soybean	Into soybean	Out of soybean
Coccinellids	0.79 ± 0.19	0.71 ± 0.26	1.88 ± 0.21	1.10 ± 0.23	2.61 ± 1.33	0.92 ± 0.43	0.67 ± 0.50	0.17 ± 0.063
Syrphids	32.60 ± 9.82	36.76 ± 7.42	149.1 ± 50.10	128.8 ± 24.61	203.6 ± 79.10	120.6 ± 46.55	50.30 ± 38.25	15.54 ± 10.67
Toxomerus marginatus	8.24 ± 1.84	16.12 ± 4.65	73.88 ± 46.64	70.87 ± 27.95	15.05 ± 5.93	25.67 ± 10.32	16.36 ± 13.12	8.27 ± 6.23
Eupeodes americanus	19.33 ± 8.88	15.38 ± 7.73	58.33 ± 8.10	50.90 ± 19.03	179.3 ± 76.35	90.34 ± 35.82	28.12 ± 22.30	5.62 ± 4.16
All other predators combined	2.02 ± 0.52	1.62 ± 0.38	1.92 ± 0.65	5.98 ± 2.68	1.52 ± 0.20	1.55 ± 0.37	1.53 ± 0.46	0.36 ± 1.78
